# Fighting Neuroblastomas with *NBAT1*

**DOI:** 10.18632/oncoscience.126

**Published:** 2015-02-18

**Authors:** Gaurav Kumar Pandey, Chandrasekhar Kanduri

**Affiliations:** Department of Medical Genetics, Institute of Biomedicine, Sahlgrenska Academy, University of Gothenburg, Gothenburg, Sweden

Children are vulnerable to extracranial solid tumours, known as neuroblastomas, at a very young age. These tumors are derived from primitive sympathetic neural precursors and they account for almost 15% of all pediatric cancer deaths (1). The most distinguishing feature of these tumors is their heterogeneous clinical behavior, which can range from spontaneous regression to highly aggressive metastatic disease. This variability in the disease outcome depends on several factors such as the age of the patient, the stage of the disease, and the genetic profile.

In the last decade, we have witnessed major advances in the field of tumor biology and in drug development for treatment of various cancers including neuroblastoma. Despite these recent diagnostic and clinical advancements, the survival rate of patients with high-risk neuroblastomas still remains less than 50 per cent (2). The poor clinical outcomes in high-risk patients compel us to improve our understanding of these tumors further and to use more innovative approaches to identify candidate genes that may contribute to the disease.

Our recent study published in Cancer Cell journal has employed one such approach, whereby transcriptomes of low- and high-risk neuroblastomas were sequenced to identify differentially expressed long noncoding RNAs (lncRNAs) with possible roles in disease pathogenesis (3). LncRNAs are untranslated transcripts more than 200bp in size, with known regulatory functions in various key biological processes that have been implicated in development and differentiation (4). We identified a set of 24 unannotated lncRNAs as being significantly differentially expressed in low- and high-risk neuroblastomas. In addition, we also identified differentially expressed lncRNAs that mapped to characteristic non-random chromosomal aberrations associated with high-risk neuroblastomas such as the *MYCN* amplified region on chromosome 2, and chromosomal deletions at 1p and 11q regions. These apparently differentially expressed lncRNA signatures might serve as biomarkers for determination of the prognosis of neuroblastomas, and their functional characterization would give major clues about the underlying biology of neuroblastoma pathogenesis.

One of the most interesting findings in our transcriptome study was the functional characterization of a lncRNA LOC729177 (known as neuroblastoma associated transcript1 or *NBAT1*; formerly referred to as *NBAT-1*) that maps to a neuroblastoma hotspot on the 6p22 region. This region has been shown to harbor three neuroblastoma associated SNPs and one of them―rs6939340―maps to intron 2 of *NBAT1* (5). This lncRNA has specific clinical and functional characteristics that make it a highly interesting molecule regarding neuroblastoma. *NBAT1* expression analysis in large datasets of 591 tumors showed that low expression is associated with poor clinical outcomes. Moreover, *NBAT1* expression can be used as an independent prognostic marker for prediction of event-free survival in neuroblastoma patients. Our study demonstrated that the lower levels of *NBAT1* in high-risk patients are due to two intricate transcriptional regulatory mechanisms involving CpG methylation and genotype at the high-risk neuroblastoma-associated SNP rs6939340. Hypermethylation of the *NBAT1* promoter and G/G genotype at the high-risk neuroblastoma-associated SNP were found to be significantly associated with lower expression of *NBAT1* in high-risk patients, suggesting that both CpG methylation and G/G genotype contribute to lower expression of *NBAT1* in high-risk patients.

**Figure:**
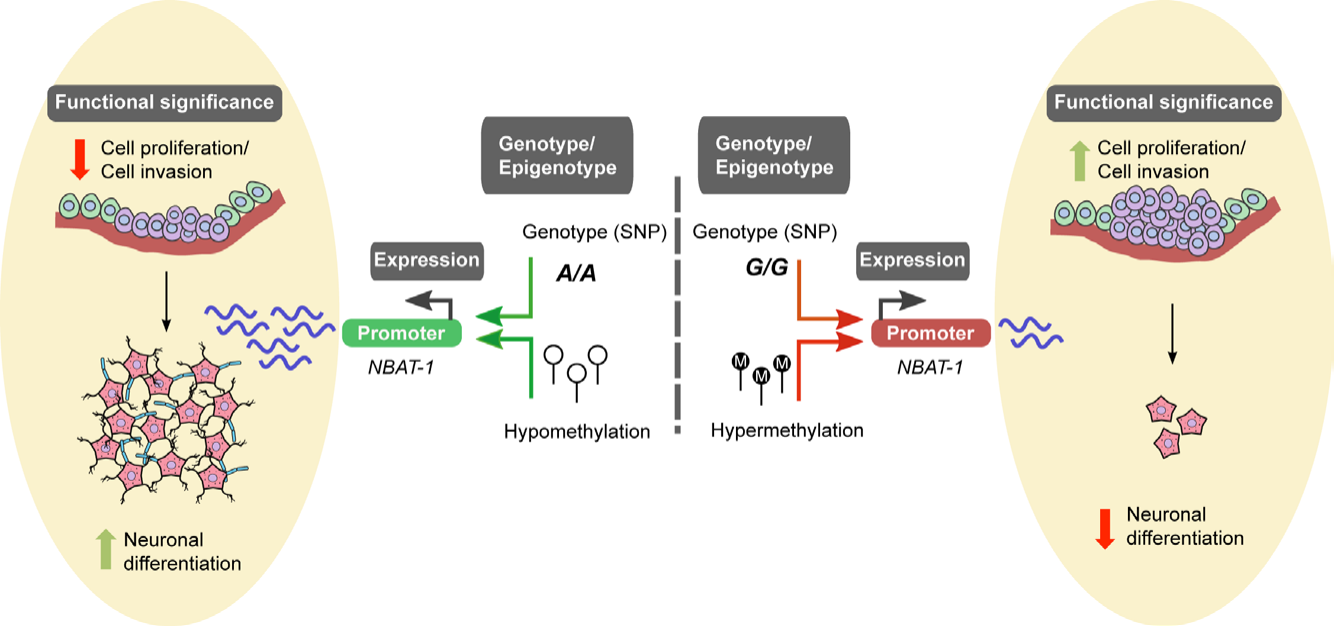
Model explaining functional role of *NBAT1* during neuroblastoma pathogenesis

Functional characterization of *NBAT1* as a novel biomarker for neuroblastoma revealed its property as a tumor suppressor, making it a highly attractive target for novel therapeutic strategies for high-risk neuroblastoma patients. *NBAT1* regulates biological processes that are highly critical for tumor development, such as cell proliferation and invasion by associating with PRC2 member, EZH2. This molecular interaction suppresses critical oncogenic networks, comprising the *SOX9*, *OSMR*, and *VCAN* genes, and affects the proliferative and invasive properties of neuroblastoma cells. The role of *NBAT1* as an effective tumor suppressor of neurobastomas complements its very important functions during neural lineage differentiation. Impaired neuronal differentiation of sympathetic neural precursors is one of the important features of high-risk neuroblastomas (6). *NBAT1* expression is upregulated during retinoic acid induced neuronal differentiation of neuroblastoma cells. Its elevated levels suppress the negative regulator of the neuronal differentiation program *NRSF*/*REST*, thus promoting proper differentiation of neural precursor cells. Based on these observations, we proposed that a higher level of *NBAT1* in low-risk tumors controls neuroblastoma progression and promotes neural differentiation. On the contrary, the hypermethylated *NBAT1* promoter and the G/G genotype at the disease associated SNP in high-risk neuroblastomas results in decreased expression of *NBAT1*, leading to highly proliferative and invasive neuroblastoma cells with an impaired ability to differentiation. This interesting correlation between lower expression of *NBAT1* and poor differentiation in high-risk tumors makes it a hotspot for therapeutic intervention.

Our observations on *NBAT1* open up novel lncRNA based treatment approaches for neuroblastomas. Furthermore, they affirm our hypothesis that lncRNAs are key molecules in determining the course of neuroblastoma disease. In conclusion, these observations have put lncRNA research in neuroblastoma disease under the spotlight and they have set the stage for further identification of novel lncRNA-based biomarkers for the risk stratification of neuroblastomas. They may also lead to druggable targets for neuroblastoma treatment.
